# Low polyethylene creep and wear following mobile-bearing unicompartmental knee replacement

**DOI:** 10.1007/s00167-020-06243-7

**Published:** 2020-09-17

**Authors:** Priyanka Ghosh, Hasan R. Mohammad, Benjamin Martin, Stefano Campi, David W. Murray, Stephen J. Mellon

**Affiliations:** Oxford Orthopaedic Engineering Centre, NDORMS, University of Oxford, Oxford, UK

**Keywords:** Unicompartmental, Unicondylar, Knee arthroplasty, Knee replacement, Radiostereometric analysis, Polyethylene

## Abstract

**Purpose:**

The Oxford unicompartmental knee replacement (UKR) has a fully congruent mobile bearing to minimise wear. However, with younger higher demand patients, wear remains a concern. The aim of this study was to quantify the wear rate of Phase 3 Oxford UKR bearings over the course of 5 years and to identify the factors that influence it.

**Methods:**

40 medial Oxford UKRs recruited for a randomised study of cemented and cementless fixation were studied with Radiostereometric analysis (RSA) at 1 week, 3 months, 6 months, 1 year, 2 years, and 5 years post-operatively and bearing thickness was calculated. Penetration, defined as the change in thickness compared to the 1-week measurement, was determined. Creep (early penetration) and wear (late penetration at a constant rate) were calculated. The influence of demographic factors, Oxford Knee Score (OKS), Tegner score, fixation and bearing overhang (determined by RSA) on wear was analysed.

**Results:**

After 6 months the penetration rate was constant, indicating that wear alone was occurring. The wear rate was 0.07 mm/year (SD 0.03). The creep was 0.06 mm with about 95% occurring during the first 3 months. There was no significant relationship between fixation (cemented/cementless), age, component size, OKS and Tegner score with wear rate. Increasing BMI was associated with decreasing wear (*p* = 0.042). 37/40 bearings overhung the tibia to some extent and 23/40 overhung the tibia medially. An increase in the area of overhang (*p* = 0.036), amount of medial overhang (*p* = 0.028) and distance between the bearing and tibial wall (*p* = 0.019) were associated with increased wear. Bearings that did not overhang (0.06 mm/year) had less wear (*p* = 0.025) than those that did (0.08 mm/year). There was no relationship (*p* = 0.6) between the femoral contact area and wear.

**Conclusion:**

During the first three to six months after implantation, the bearing becomes 0.06 mm thinner due to creep. The combined wear rate of the upper and lower surfaces of the bearing is constant (0.07 mm/year). The wear is lower if the bearing does not overhang the tibia so surgeons should aim for the bearing to be close to the tibial wall. The orientation of the femoral component does not influence wear.

**Level of evidence:**

Retrospective Study, Level III.

## Introduction

Polyethylene wear is a significant concern following unicompartmental knee replacement (UKR) as it is important to use thin bearings to preserve bone. Linear wear can potentially cause catastrophic failure with bearing wear through or fracture. Marked linear wear may also result in deterioration of kinematics and abnormal implant loading with loosening and patellofemoral joint problems. Although wear particle-induced osteolysis secondary to volumetric wear can be an issue this usually only happens if there is marked linear wear [1, 19]. Therefore, it is most important to minimise linear wear [3].

Unicompartmental knee replacements are either fixed bearing or mobile bearing. The mobile bearing of the Oxford UKR is fully congruent with a spherical femoral component above and a flat tibial component below. This design maximizes contact area, minimises contact stress, and theoretically minimises wear [18, 26]. There is some debate, based on wear simulator studies, about whether lower-conforming fixed bearing UKRs have more or less volumetric wear than mobile-bearing devices, however, mobile-bearing devices have lower linear wear rates due to the higher contact areas [4, 14, 17]. There is little in vivo data about the linear wear rate of fixed-bearing UKRs: In a radiographic study at a mean of 5 years, wear of between 1 and 7 mm was measured in about 30% of cases [28]. A retrieval study found annual wear of 0.15 mm/year, however, this was probably a best case estimate as many bearings that failed catastrophically due to wear were not retrieved or were unmeasurable [2]. In contrast, previous studies of the mobile bearing Oxford UKR have reported mean in vivo bearing wear rates of between 0.02 and 0.07 mm/year [12, 13, 22, 23]. Proposed explanations for variation in mobile-bearing wear rate include degree of impingement on bone or cement, degree of medial overhang [9], and differences in the polyethylene manufacturing process (machined vs. moulded) [16]. There is also some evidence that the minimally invasive Phase 3 Oxford, despite improved functional outcomes, has an increased wear rate.

Radiostereometric analysis (RSA) is the gold standard for measuring in vivo bearing wear. It accurately captures the relative position of the metallic tibial and femoral components such that bearing thickness can be determined. Based on the decrease in thickness over time the wear rate can be estimated. However, early after implantation there is plastic deformation (creep) of the bearing [18, 26]. Therefore, a single RSA measurement cannot accurately determine the wear rate as the relative contribution of creep and wear to the decreased thickness of the bearing cannot be determined. Furthermore, the precise thickness of the bearing after implantation is not known. The solution is to take an RSA measurement immediately after implantation to assess the initial thickness, then serial measurements to distinguish creep and wear. There is uncertainty as to how much creep occurs after Oxford UKR. A recent RSA study concluded there was no appreciable creep, whereas previous wear simulator studies have shown marked creep during the first million cycles [9].

With the average age of knee replacement patients decreasing and life expectancies and activity levels increasing, measurements of in vivo bearing wear and exploration the factors that affect it are important. The aim of this study was to determine the in vivo polyethylene wear rate of the minimally invasive Phase 3 Oxford medial UKR using RSA. To determine the wear rate accurately and to distinguish creep from wear, RSA measurements were taken immediately postoperatively and at a series of time points over the course of 5 years [6]. The wear rate was tested for associations with the method of fixation (cemented vs. cementless), presence of bearing overhang, femoral contact area, body-mass index (BMI), patient age, Oxford Knee Score (OKS) and Tegner activity score and component size (tibial or femoral).

## Methods

The study was approved by the Regional Ethics Committee (C02.101, contact person: jo.franklin@orh.nhs.uk). It was conceived as part of the early assessment of a new cementless Oxford UKR, with the primary aim to compare the two-year implant migration of the cementless to the cemented Oxford UKR in a randomised study using RSA [11].

**Fig. 1:**
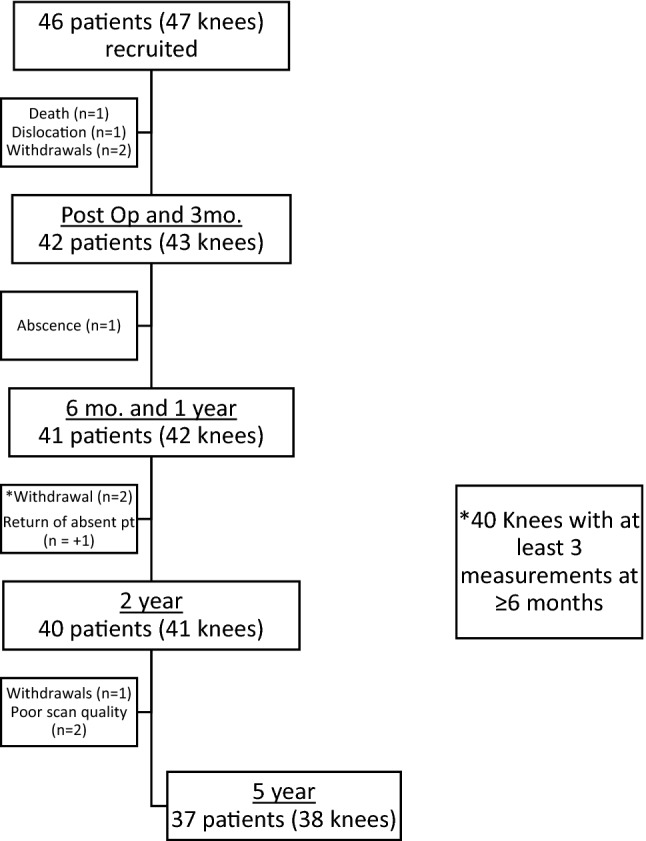
Study population over time

Forty-six patients (47 knees) were prospectively recruited and their informed consent was obtained. All patients received a Phase 3 medial Oxford UKR (Zimmer Biomet, Warsaw, IN, USA) between October 2008 and March 2010 at the Nuffield Orthopaedic Centre in the United Kingdom. Twenty-three received cementless and twenty-four received cemented components. Patients were eligible for inclusion in the study if they underwent primary medial Oxford UKR using the recommended indications [8]. These have been well described and consist of bone-on-bone medial compartment osteoarthritis, full-thickness lateral cartilage, functionally intact anterior cruciate and medial collateral ligaments, and the absence of bone loss on the lateral facet of the patella. Due to a few exclusions and withdrawals, small variations in sample size existed at different time points (Fig. 1). 42 patients (43 knees) were included at the beginning of the RSA study. The mean age of the cohort was 67 years (SD 8), mean BMI was 30 (SD 6), and mean pre-op OKS was 24 (SD 6). The cohort was composed of 21 cemented and 22 cementless knees. Figure 1 and Table 1 detail how many knees were available for analysis of linear penetration at each time point. The bearing thickness measured immediately post-operatively was 0.61 mm (SD 0.15) thicker than the nominal bearing thickness.

**Table 1:** Mean bearing penetration over time, calculated using post-op bearing thickness for reference

Months	3	6	12	24	60
Count	40	40	40	40	37
Mean penetration (mm)	0.08	0.101	0.131	0.208	0.419
Std. dev	0.119	0.105	0.102	0.124	0.166
95% CI	0.038	0.034	0.033	0.040	0.055

### Analysis

Patients had stereo radiographs at each of the follow-up time points: 1-week post-op (reference), 3 months, 6 months, 1 year, 2 years and 5 years. Radiographs were taken while the patients stood within a calibration frame in weight-bearing, full knee extension position as previously described [25]. Model-based RSA software (Medis Specials, Leiden, The Netherlands) was used to determine the position and orientation of the femoral and tibial components in three-dimensional space. The closest linear distance between components was then calculated using Matlab (The Math Works Inc., Natick, Massachusetts). This was taken to be the bearing thickness [27] and was determined for each knee at each time point. Linear penetration was then calculated for each knee at 3 months, 6 months, 1 year, 2 years and 5 years by subtraction from the immediate post-operative bearing thickness.

In the early post-operative period, linear penetration is a combination of both creep and wear [5, 24]. Once the penetration rate becomes constant, penetration can be assumed to be purely due to wear. Mean, standard deviation and 95% confidence limits were calculated for linear penetration at each time point and were plotted to determine when creep and wear occurred. The graph was found to be linear from 6 months onwards, indicating that the penetration rate was constant and wear alone was occurring. Regression was used to find a linear penetration rate for each knee and the gradient of this line was considered to be the wear rate in mm/year. Creep was determined by subtracting the calculated wear from the total penetration at each time point. Knees were included in statistical analyses if they had wear rates derived from regression of at least 3-time points. This resulted in the exclusion of three knees leaving a maximum of 40 for analysis. Mean and standard deviation were calculated for wear rates.

Unpaired *t* tests were used to determine whether significant differences in the wear rate existed by the method of fixation (cemented vs. cementless). Pearson Correlation was used to test the association of wear rate with BMI, age, 5-year Oxford Knee Score (OKS) and Tegner activity score. The association of component size (tibial or femoral) with wear rate was tested with an ANOVA.

**Fig. 2:**
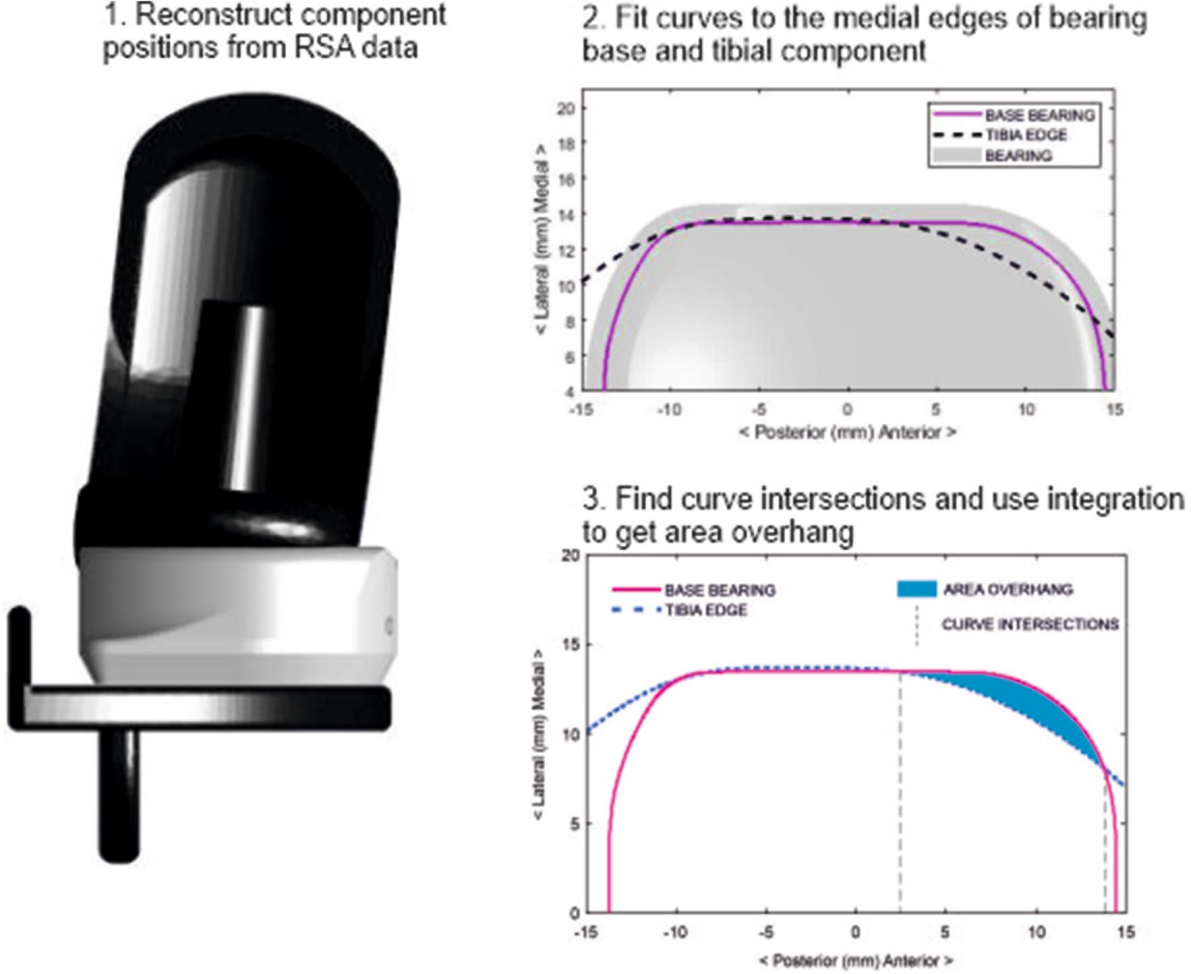
Method for calculating the area of bearing overhang from RSA data. The bearing can exhibit no medial overhang in the coronal plane [9] but overhang at the anterior or posterior margins of the tibial component is still possible. Both Medial Overhang and Area Overhang calculations are done with the assumption that the bearing is parallel with the wall of the tibial component

Bearing overhang beyond the tibial component and bearing distance from the tibial vertical wall were determined using the femoro-tibial contact point, as the bearing is not visible on stereo-radiographs. The femoro-tibial contact point was defined where joint space was narrowest and reflects the centre position of the bearing due to the spherical design of the femoral component and the fully congruent bearing. The bearing was assumed to be parallel with the tibial wall. Medial bearing overhang was noted when the distance between the femoro-tibial contact point and the medial edge of the tibial component was less than the distance from the centre to the medial edge of the bearing [9]. The area of bearing overhanging the medial edge was found by fitting curves to the medial edges of the bearing base and the tibial component. If these curves intersected, they were integrated between the intersection points to find the area between the curves (Fig. 2). The distance from the lateral edge of the bearing to the vertical tibial wall in millimetres was also calculated. If this was found to be negative the bearing was considered to be impinging on the wall.

The area of the femoral component in contact with the upper surface of the bearing was also estimated. This was done by making the centre of the sphere that intersects with the upper surface of the bearing the origin of a spherical coordinate system, defining the border of the bearing surface (azimuth and elevation, in radians) and finding the femoral component coordinates that lay within this border. The area of the upper surface of the bearing occupied by the femoral component in radians squared was converted to millimetres squared using the radius of the sphere. As with the estimation of tibial overhang, this was done with an assumption that the bearing was parallel with the wall of the tibial component.

Unpaired *t* tests were used to determine whether significant differences in the wear rate for presence or absence of medial overhang. Pearson Correlation was used to test the association of wear rate with medial overhang (mm), area overhang (mm^2^) and femoral contact area (mm^2^). A *p* value of ≤ 0.05 was considered significant.

## Results

**Fig. 3:**
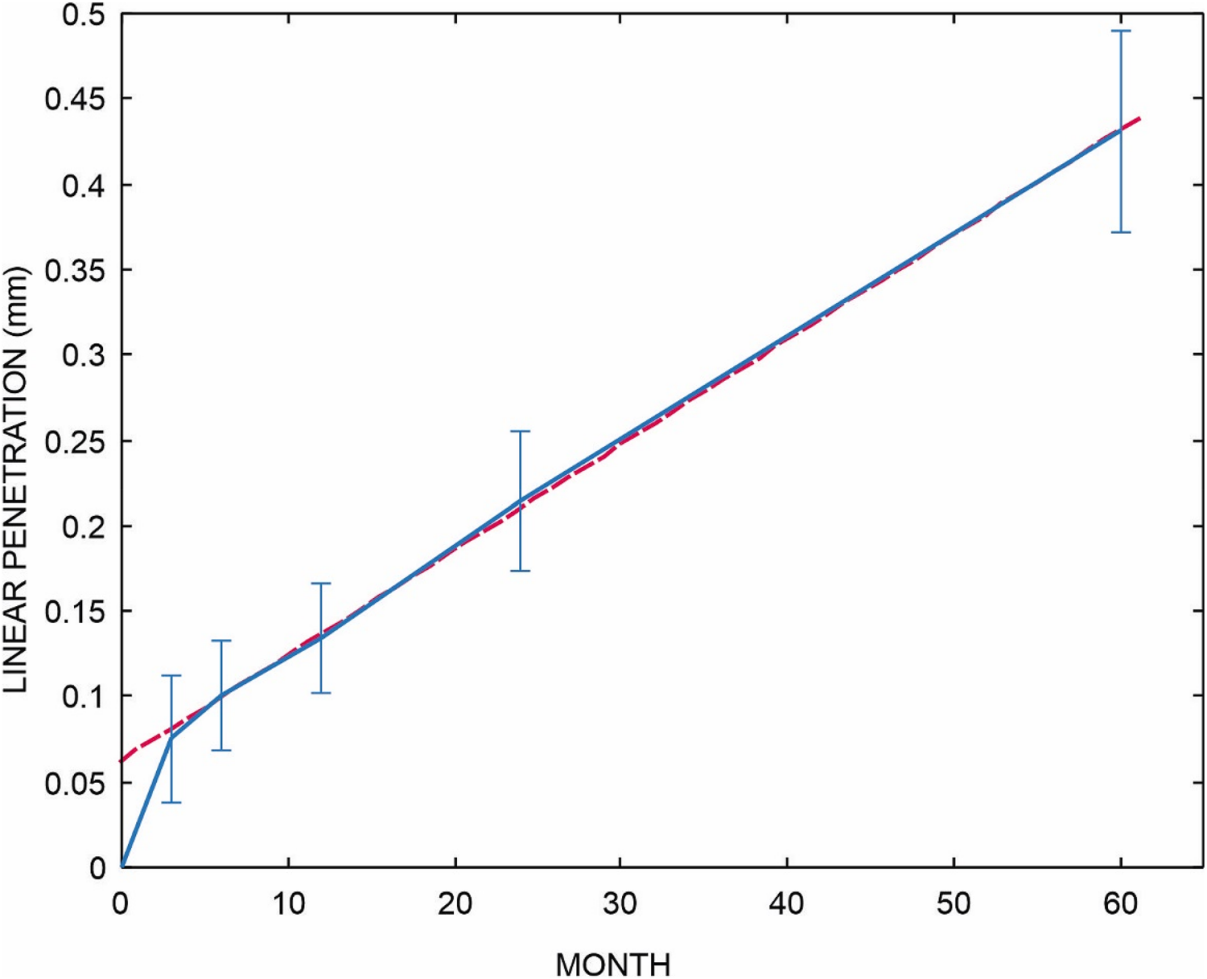
Mean linear penetration in blue with 95% confidence intervals. Linear regression between months 6, 12, 24 and 60 is overlaid in red

**Fig. 4:**
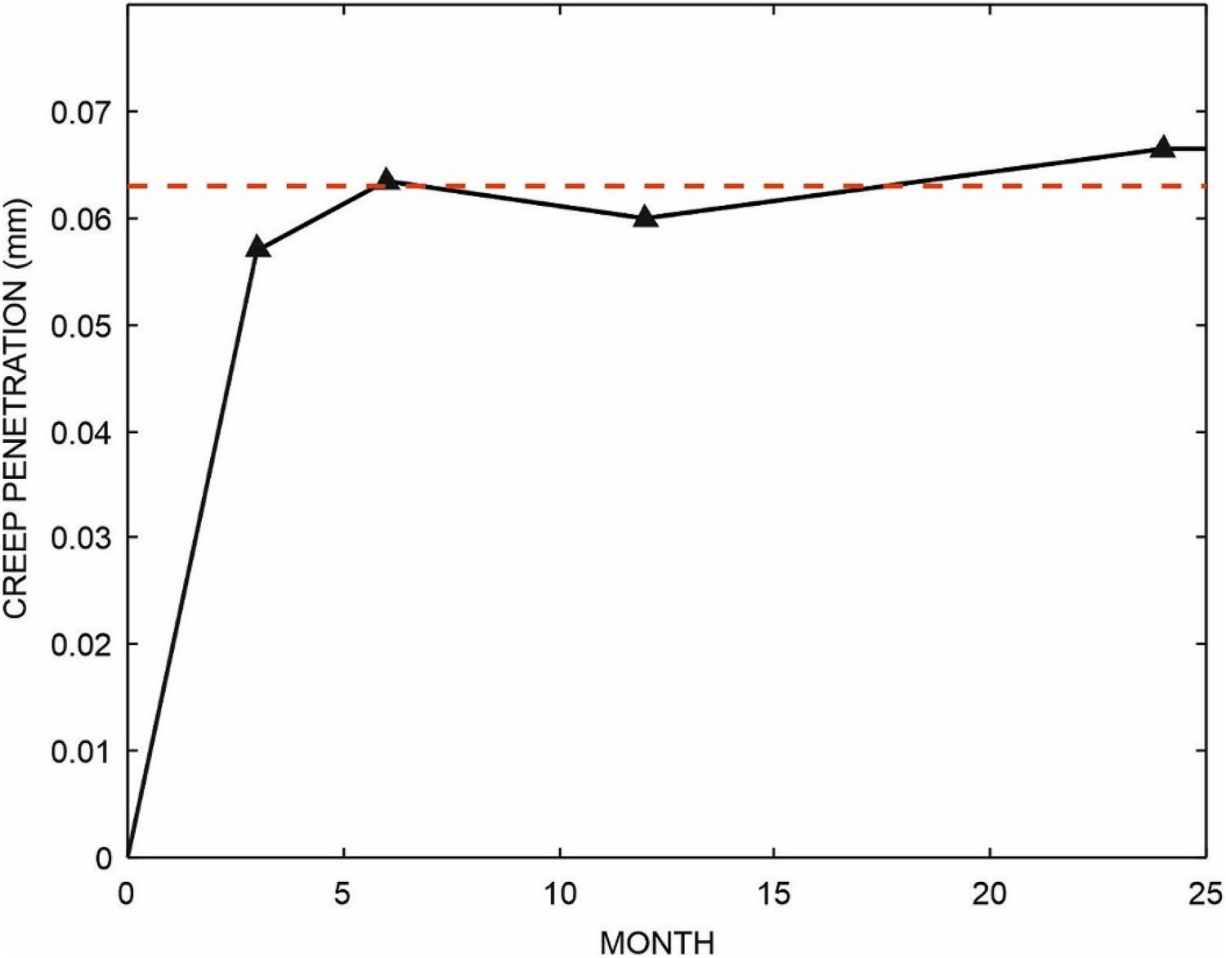
Creep penetration calculated by subtracting wear penetration from overall penetration at each time point. The red dotted line indicates the total creep penetration

The linear penetration versus post-operative time (Fig. 3; Table 1) was a straight line from six months onwards, indicating that the penetration rate was constant and there was wear alone and no creep. The mean linear wear rate, was 0.071 mm/year (SD 0.032). The mean penetration due to creep, (i.e. the regression line intercept with the Y axis) was 0.063 mm (SD 0.10). 95% of creep had occurred at 3 months. Penetration attributable to creep had ended by 6 months (Fig. 4).

No significant difference (*p* = 0.74) in linear wear rate was found between cemented and cementless knees (Table 2). There was no significant relationship between OKS and linear wear (*p* = 0.351). There was also no significant relationship between Tegner score and linear wear (*p* = 0.91). Patient BMI had a statistically significant relationship with wear rate (*p* = 0.042), which suggested that for every 5-point increase in BMI the wear rate decreased by 0.01 mm/year. There was no significant relationship between the size of either the tibial component or femoral component and the wear rate. There was no significant relationship between patient age and wear rate.

**Table 2:** Comparisons of linear wear rate between groups

Group (*n*)	Mean wear rate (SD) (mm/year)	Association
Cemented knees (19)	0.072 (0.035)	*p* = 0.74
Cementless knees (21)	0.076 (0.039)	
Medial overhang (23)	0.082 (0.033)	*p* = 0.055
No overhang (17)	0.063 (0.028)	
Area overhang (37)	0.076 (0.033)	*p* = 0.025*
No area overhang (3)	0.058 (0.007)	
Tibia size: A (5)	0.073 (0.029)	*p* = 0.94
Tibia size: B (8)	0.067 (0.047)	
Tibia size: C (14)	0.076 (0.033)	
Tibia size: D (3)	0.085 (0.023)	
Tibia size: E (7)	0.080 (0.023)	
Tibia size: F (3)	0.062 (0.020)	
Femur size: small (16)	0.072 (0.040)	*p* = 0.95
Femur size: medium (12)	0.075 (0.021)	
Femur size: large (12)	0.076 (0.030)	

*p < 0.05

**Fig. 5:**
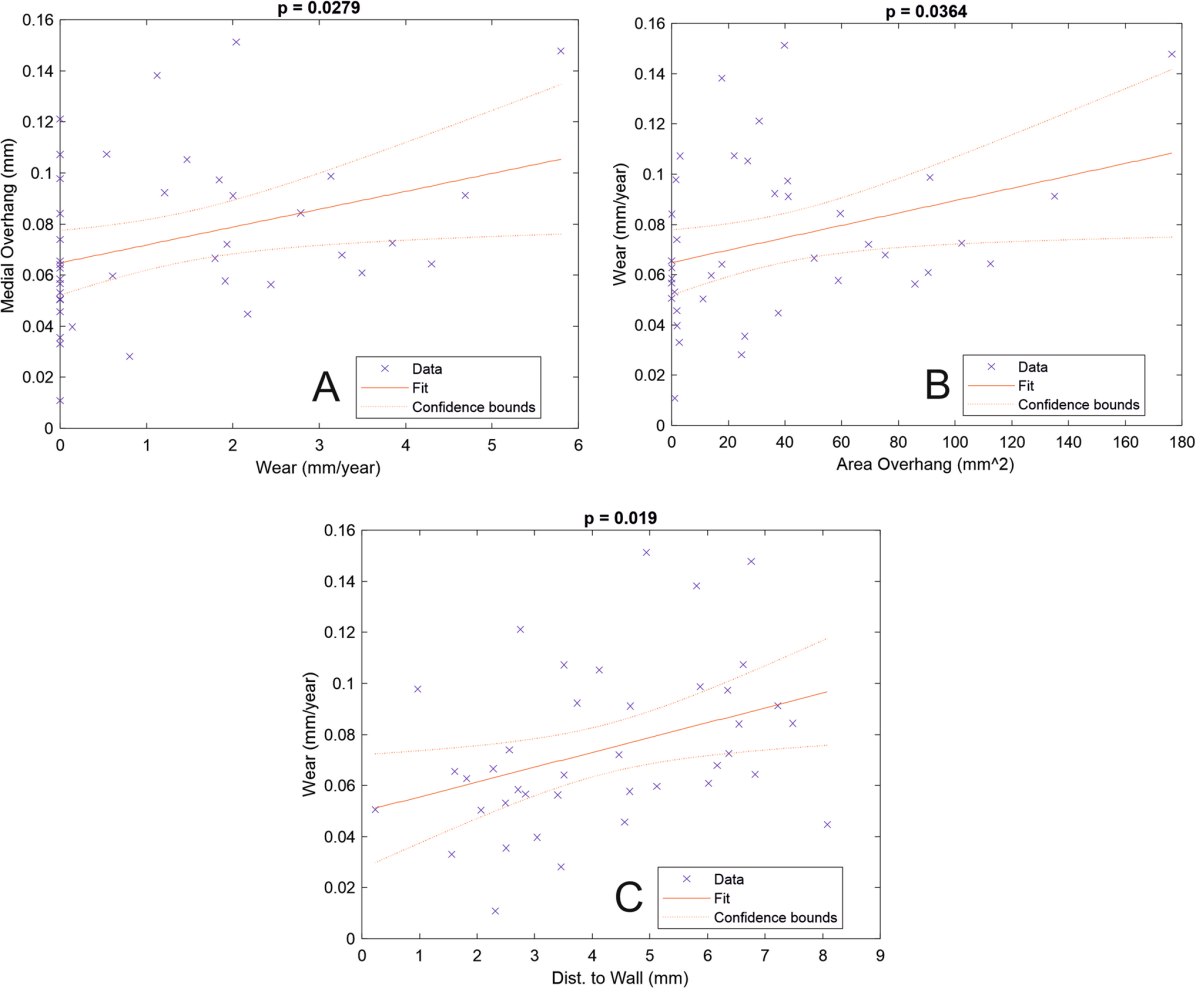
The association between linear wear rate and various aspects of bearing position. **a** The association with medial overhang was statistically significant (*p* = 0.0279). **b** The association with area overhang was significant (*p* = 0.0364). **c** The association with the distance of the bearing to the tibial wall was significant (*p* = 0.019)

Twenty-three out of 40 knees had a medial overhang. Although the medial overhang cohort had a higher wear rate than the non-overhang cohort, the difference was shy of statistical significance (*p* = 0.055) (Table 3). However, the amount of medial overhang, calculated with those not overhanging considered to have zero overhang (Fig. 5a), was significantly related to wear rate (gradient 0.007, *p* = 0.0279). This analysis suggests that for every millimetre of medial overhang the wear rate increases by 0.007 mm/year. If only the knees with bearings overhanging medially are considered, the relationship is not statistically significant (*p* = 0.28) but the gradient is similar (0.005 mm/year).

**Table 3:** Associations with linear wear rate

Variable (*n*)	Mean (SD)	Gradient vs. wear (mm/year)	Association
Age (40)	66.5 (8.3) Years	0.001	*p* = 0.12
BMI (37)	30.2 (6.6)	− 0.002	*p* = 0.042*
OKS at 5 years (39)	39.1 (9.6)	0.0005	*p* = 0.351
Tegner score at 5 years (25)	2.72 (1.37)	0.0005	*p* = 0.91
Medial overhang: all (40)	0.73 (2.22) mm	0.005	*p* = 0.045*
Medial overhang: overhangers only (23)	2.32 (1.43) mm	0.005	*p* = 0.28
All patients [underhangers = 0] (40)	0.07 mm/year wear (0.032)	0.007	*p* = 0.028*
Area overhang: overhangers only (37)	40.8 (43.1) mm^2^	0.006 mm/year/30 mm^2^	*p* = 0.064
Area overhang: all (40)	37.7 (42.8) mm^2^	0.0002	*p* = 0.036*
Femoral contact area (40)	518.2 (89.0) mm^2^	− 0.00003	*p* = 0.603
Distance to tibial wall (40)	4.20 (2.02) mm	0.006	*p* = 0.019*

*p < 0.05

In the thirty-seven knees that exhibited area overhang, the average wear rate was significantly (*p* = 0.025) greater than in the 3 that did not overhang (Table 2). When all 40 patients were analysed, and those without overhang were considered to have 0 mm^2^ of overhang, there was a statistically significant (*p* = 0.036) relationship between area overhang and linear wear rate (Table 3), suggesting that for every 30 mm^2^ of bearing overhanging the tibial component the wear rate increased by 0.006 mm/year (Fig. 5b).

The mean distance from the lateral edge of the bearing to the vertical wall of the tibial component was 4.20 mm (SD 2.02). Only 2 bearings were considered to be impinging on the wall. The distance between the bearing and the tibial wall had a statistically significant (*p* = 0.019) relationship with linear wear rate (Fig. 5c). For every increase in this gap by 1 mm the wear rate increased by 0.006 mm/year.

The average area of contact between the femoral component and bearing was found to be 518.2 mm^2^ with a wide variability (SD 49 mm^2^). However, there was no significant relationship between the area of contact and the wear rate.

## Discussion

The most important finding of the study is that the average linear wear rate (0.07 mm/year) and early creep (0.06 mm) of the Phase 3 Oxford UKR bearing as it is currently being used is low. The wear rate was the same with cemented and cementless components and was not related to age, component size, OKS and Tegner activity score. There was a relationship between BMI and bearing overhang with wear rate.

This is the first in vivo study to distinguish wear and creep after knee replacement, in particular, after mobile-bearing UKR. The average amount of creep was 0.06 mm and most of this occurred within the first three months. Creep appeared to be complete by 6 months. Thereafter, wear occurred at a constant rate. Surprisingly, creep was not found in an RSA study of the Oxford UKR by Horsager et al. but this may be because the current study had earlier RSA assessments. RSA studies by Glyn-Jones et al. [7] and Campbell et al. [5] of total hip replacement acetabular liners have also clearly demonstrated early creep in addition to ongoing wear at a constant rate.

The 0.07 mm/year linear wear rate calculated in this study is low, similar to previously reported in vivo wear rates for the Phase 3 Oxford UKR. It is also substantially lower than that reported for fixed bearing studies [2, 28]. However, it is higher than the previous RSA wear measurement of 0.02 mm/year in Phase 2 Oxford bearings [13]. It is not clear why this is. Although medial overhang increases wear, the bearings without medial overhang still had higher wear than Phase 2 bearings (0.06 mm/year). Impingement against bone or cement increases wear but this is unlikely to be the explanation as the surgeons involved with the study took care to avoid impingement. It may relate to the higher function achieved with the minimally invasive approach. Alternatively, it may relate to operative errors resulting from the restricted view with the original Phase 3 instrumentation and minimally invasive approach. If this is the case it should be addressed with the new Microplasty instrumentation, which was introduced after this study and makes the operation simpler and more reliable.

Bearing fracture is a rare complication but the risk increases if there is both marked oxidation and wear of the polyethylene, particularly if the bearing wears down to less than 2 mm thick [20, 21]. Therefore, to minimise this risk of fracture in the third decade after surgery, surgeons should aim to use 4 mm rather than 3 mm bearings in young patients, unless they are small and there is need to conserve bone. Furthermore, consideration should be given to the use of an irradiated vitamin E polyethylene as this should decrease both wear and oxidation.

It was found that increasing medial bearing overhang was associated with an increased linear wear rate of 0.007 mm/year per millimetre overhang. This is less than that reported by Horsager et al. (0.015 mm/year per mm) [9]. In addition, an increase in the area by which the bearing overhung the tibia was associated with an increased linear wear rate of 0.006 mm/year per 30 mm^2^ overhang. As 37 bearings had area overhang and only 23 had medial overhang it suggests that any overhang, not just medial, is important. However, it is not clear whether anterior, posterior or medial overhang is most important. Each millimetre of medial overhang decreases the contact area on the lower bearing surface by about 30 mm^2^. Not surprisingly, therefore, an increase in medial overhang by 1 mm and an increase in area overhang of 30 mm^2^ increases the wear rate by a similar amount (0.007 mm/year and 0.006 mm/year, respectively). This increased overhang is about 2.5% of the whole contact area of the bearing. A simplistic analysis of wear would suggest that the volumetric wear is independent of contact area and, therefore, that linear wear is inversely related to the contact area. A decrease in the contact area of 2.5% increased the wear rate by over 10%, this suggests that the increased wear due to overhang is not simply due to the decreased contact area. With overhang the bearing may rotate as it moves backwards and forwards during activity, resulting in cross shear which is known to markedly increase polyethylene wear [10]. This rotation would be minimised if the bearing was close to the wall, perhaps explaining in part the association between the distance the bearing is from the wall and wear rate.

Whatever the reason for the increased wear surgeons should try and minimise bearing overhang. As large a tibia as possible should be implanted. This is achieved by making the vertical tibial cut just medial to the apex of the spine. Similarly, surgeons should avoid implanting an unnecessarily large femur, as the size of the bearing is the same as the size of the femur. The closer the bearing is to the wall the lower the wear rate. To avoid the bearing hitting the wall surgeons should aim for the bearing to be about one millimetre from the tibial wall. This is best achieved by having the foot of the Microplasty drill guide resting against the vertical tibial cut when the femoral drill holes are made and will result in the femoral component being central or slightly lateral to central in the femoral condyle. The bearing should also track parallel to the wall, which is best achieved by directing the vertical tibial cut towards the anterior superior iliac spine. The area of contact between the femoral condyle and the bearing was not related to wear, so the alignment of the femoral component does not influence wear.

There have been concerns that polyethylene wear might be higher with cementless rather than cemented fixation. We found no significant difference in wear rate between cemented and cementless components and notably the current study is a randomised comparison of cemented and cementless UKR. Surprisingly, decreased wear was associated with increased BMI. Although this is plausible, as patients with a lower BMI tend to be more active, we have not seen this in previous RSA wear studies so we cannot be certain if it is a real association [16]. Similar to our previous studies, no relationship was found between age, OKS, Tegner score and wear rate. In this current study, with compression moulded polyethylene bearings, there was no relationship between component size and wear. In our previous study with the same bearing there was no relationship between component size and wear but there was a relationship for machined bearings [16].

In most previous RSA wear studies of the Oxford UKR, bearing wear was determined by subtracting the RSA measured bearing thickness at a certain post-operative time from an estimate of bearing thickness at implantation. These estimates have been made by measuring unused bearings after implantation or using information from other RSA studies of bearing thickness immediately after implantation [13, 23]. This approach is not as accurate as the one used in the current study, in which the thickness of each bearing was measured with RSA immediately after implantation. Furthermore, it tends to overestimate wear as it ignores the effects of creep. In the current study, we found the bearing thickness at implantation was 0.61 mm thicker than the nominal bearing thickness, similar to previous estimates [13], with the early creep of 0.06 mm. So, future studies will obtain a more accurate estimate of wear using nominal size plus 0.55 mm (0.61–0.06) as bearing thickness at implantation.

The main limitations of the study relate to the measurements of bearing overhang. We made two assumptions: first, that the bearing lies parallel to the wall, even though if it is not close to the wall it probably rotates. Second, that the position of the bearing in extension, as when RSA X-rays are taken, is similar to the position during activity, although we know some roll-back is occurring [15]. Despite these assumptions, it was found that however overhang was analysed it was related to wear, so it is likely to be a true observation. Another limitation is that wear was estimated over a short period of 4.5 years. However, as the average wear rate was 0.07 mm/year, the total wear was about 0.3 mm. This is appreciably larger than the accuracy of our RSA system, which is about 0.1 mm when assessed with repeated X-rays [13]. Another limitation was that we were not able to analyse all the stereo radiographs from all patients at all time points. This was partly because some patients withdrew due to poor health or death and partly because some stereo radiographs were of poor quality so could not be analysed. This is unlikely to have influenced the results.

## Conclusion

The early creep (0.06 mm) and combined wear rate (0.07 mm/year) of the upper and lower surfaces of the bearing is low. As the wear rate is constant surgeons should aim to use 4 mm rather than 3 mm bearings in young patients with long life expectancy, unless there is a need to conserve bone. The wear rate was not related to age, activity, fixation, component size or femoral component orientation. However, the wear is lower if the bearing does not overhang the tibia so surgeons should also aim for the bearing to be close to the tibial wall.
